# HealthCare educational differences in pain management, adverse childhood experiences and their relationship to substance use disorder education

**DOI:** 10.1186/s13011-022-00436-8

**Published:** 2022-02-07

**Authors:** Myriam Shaw Ojeda, Aleda M. H. Chen, Tessa Miracle, Elizabeth Delaney, Caroline E. Freiermuth, Jon E. Sprague

**Affiliations:** 1 Ohio Pharmacists Association, Columbus, OH USA; 2 Cedarville University School of Pharmacy, Cedarville, OH USA; 3 The Ohio State University College of Pharmacy, Columbus, OH USA; 4 Member of the Ohio Attorney General’s Scientific Committee for Opioid Prevention and Education (SCOPE), Columbus, USA; 5 Cedarville University School of Nursing, Cedarville, OH USA; 6 Department of Emergency Medicine, University of Cincinnati College of Medicine, Cincinnati, OH USA; 7 Center for Addiction Research, University of Cincinnati College of Medicine, Cincinnati, OH USA; 8 The Ohio Attorney General’s Office, Columbus, OH USA; 9 The Ohio Attorney General’s Center for the Future of Forensic Science, Bowling Green State University, Bowling Green, OH 43403 USA

**Keywords:** Opioid Use Disorder, Healthcare education, Prevention

## Abstract

**Background:**

In order to assist the State of Ohio in the United States in addressing the opioid epidemic, the Ohio Attorney General appointed experts in a variety of academic disciplines to the Scientific Committee on Opioid Prevention and Education (SCOPE). The focus of SCOPE is the application of scientific principles in the development of prevention and educational strategies for reducing substance use disorder (SUD). One area of focus for SCOPE was SUD education of healthcare professionals. The objective of the present was to identify the content and extent to which future healthcare professionals are trained in pain management, SUD, and adverse childhood experiences (ACEs).

**Methods:**

In December of 2019, a survey was distributed to 49 healthcare professional schools in Ohio that included the following disciplines: medicine, pharmacy, advanced practice registered nurse (APRN), physician assistant, dentistry, and optometry. The survey included four domains: initial screening of patients, training in SUD, training in care for patients at high risk for SUD, and education in evaluating patients for ACEs. Descriptive statistics were calculated.

**Results:**

Thirty one of the forty-nine schools completed the survey. Most disciplines indicated that some form of basic training in the principles of SUD were taught in their core curriculum. The training on ethical issues surrounding SUD were not as widely covered (range 0-62.5%). Medicine, APRN, physician assistant, and pharmacy schools had a “moderate” to “great” extent of pharmacologic therapy curriculum integration. Other pain management strategies were “somewhat” to “moderately” integrated. There were variations seen in training on risk of medication misuse based on various contributors to health. At least 67.7% of medicine, APRN, physician assistant, and pharmacy programs included motivational interviewing training. The extent to which schools integrated education regarding ACEs into their curriculum varied from 0 to 66.7%.

**Conclusions:**

The study finding suggests a need for a unified, consistent, and expanded training requirement in the foundations of pain management, SUD, and ACEs in professional healthcare education.

## Introduction

During the COVID19 (SARS-CoV-2) pandemic, more than 100,000 people died in the United States as a result of drug overdose in the 12 month period ending in April 2021 [4]. In 2018 alone, drug overdose deaths totaled 46,802 in the United States, and of these deaths, the majority (70%) involved an opioid [10]. In 2017, nearly half of opioid related deaths involved a prescription opioid [10]. America is suffering from the public health crisis of prescription drug misuse, with more than 10 million Americans reporting non-medical use of opioids [14]. While the impact of opioid related deaths has been felt across the nation, the burden of this public health concern has been magnified in the state of Ohio, ranking second in the United States for drug overdose rates [31]. More specifically, Ohio has seen a 169% increase in unintentional drug overdose deaths between 2010 and 2017 [27]. After the peak in unintentional overdose deaths in 2017, there was an initial decrease in overdose deaths in 2018 but that trend has subsequently been reversed [37]. In response, Ohio has utilized several strategies that aim to prevent prescription drug misuse including the CDC prescribing guidelines, prescription drug monitoring program (PDMP), and community-based prevention efforts [5]. A focus on prescriber education is a next logical step. Without streamlined educational practices that support identification of patient need and risk, as well as alternative pain management strategies, there is a risk for biased prescribing (undertreatment of high-risk and marginalized patient groups), inconsistent pain management, unnecessary patient burden, and even possibly death [16, 18, 28].

A recent report called for reforms in substance use disorder (SUD) education with a primary emphasis on evidence-based approaches to prevention, identification, and treatment [34]. More importantly, this call identified the “silo” of curricular content as problem areas in training health professionals in SUD. A New York Times review identified that comprehensive SUD training is rare in medical education [15]. In their scoping review of SUD education, Muzyk et al. [24] found that interprofessional SUD programs improved health professional knowledge, skills, and attitudes toward SUD.

In addition to the well-known predictors of the development of opioid use disorder (OUD), such as prior substance use, mental health diseases, and concomitant use of psychiatric medications or benzodiazepines [6], the correlation between childhood trauma and both behavioral and medical issues in adulthood was demonstrated in a large, cross-sectional study by Kaiser Permanente and the Centers for Disease Control and Prevention (CDC), known as the Adverse Childhood Experiences (ACEs) Study [9]. The long-term impact of ACEs extends beyond typical markers of health like blood pressure, heart disease, or obesity and are positively correlated to risky health behavior [7]. Patients who have experienced ACEs are significantly more likely to participate in behaviors such as physical inactivity, smoking, or overeating and are disproportionately represented in patients with SUD [29]. More specifically to this cause, data from the Behavioral Risk Factor Surveillance System suggest a positive association between ACEs and prescription opioid misuse outcomes in adulthood [21].

To assist the State of Ohio in the United States in addressing the opioid epidemic, the Ohio Attorney General appointed experts in a variety of academic disciplines to the Scientific Committee on Opioid Prevention and Education (SCOPE). The focus of SCOPE is the application of scientific principles in the development of prevention and educational strategies for reducing substance use disorder (SUD). One area of focus for SCOPE was SUD education of healthcare professionals. The objective of the present study was to identify the content and extent to which future healthcare professionals are trained in pain management, SUD, social determinants of health, and ACEs in relationship to SUD.

## Methods

The Ohio Attorney General’s Scientific Committee on Opioid Prevention and Education (SCOPE) committee charged the educational sub-committee to create a survey to send out to all Ohio healthcare academic programs. The Institutional Review Board (IRB) at Cedarville University determined that this study had an exempt status.

### Study design

In December of 2019, healthcare academic program directors at Ohio universities were surveyed utilizing a modified version of the 10 core competencies outlined in the three prevention domains of the Massachusetts study of medical schools [3]. An additional emphasis was placed on ACEs training based the association of ACEs and SUD [7, 29]. The inclusion criteria for healthcare schools was accredited schools that train doctors, dentists, APRN, optometrists, physicians assistants and pharmacists. These professions were chosen as each profession is involved in the prescribing and dispensing of opioid medications for patients. In Ohio, optometrists have limited prescribing authority for opioids. Other healthcare professions that may indirectly contribute to opioid usage such as veterinarians were excluded as their practice does not directly associate with human patients. The survey covered the following topic areas: education on OUD and SUD; extent of education; education on assessment, evaluation, and treatment; education on ACEs; education on social determinants of health. Information on factors that can impact pain and how pain is perceived were also surveyed, including biologic factors (age) or societal factors (cultural beliefs). Prior to administration, the survey was pretested by the (SCOPE) committee. The membership of SCOPE consisted of nine researchers from multiple Ohio academic institutions with expertise in the areas of medicine, pharmacy, APRN, data analytics, epidemiology, toxicology, public health, behavioral economics, and cultural anthropology.

The online survey was distributed using Qualtrics software. The surveys were sent to the deans of each school who were asked to pass on the survey to individuals charged with curriculum development as it related to SUD training. Reminders regarding survey completion were given on three separate occasions. Email and phone calls were utilized to encourage the academic programs to participate in the survey.

### Data analysis

Data were analyzed in SPSS v. 25.0 (IBM) using descriptive statistics. Categorical variables were analyzed using frequencies (number, percentage). Likert-type variables were analyzed for normality using a Shapiro-Wilk test. Because the variables were not normally distributed, the data were analyzed using median and interquartile ranges.

## Results

Surveys were sent to a total of 49 schools: 7 medical, 7 pharmacy, 2 dentistry, 1 optometry, 10 physician assistant and 22 APRN. The response rate for each discipline was: Medicine 100% (*n*=7), Pharmacy 100% (*n*=7), APRN 27% (*n*=6), Physician Assistants 80% (*n*=8), Dentistry 100% (*n*=2), and Optometry 100% (*n*=1). In total, 31 of the 49 schools (63.2%) completed the survey.

Overall, most disciplines (medicine=100%; APRN, pharmacy, and physician assistants=83-87.5%) indicated that some form of basic training in the principles of SUD were taught in their curriculum, including the neurobiology of SUD as part of the core curriculum (see Table 1). Dentistry and optometry did not cover these topics. However, the training on ethical issues surrounding SUD were not as widely covered in the curriculum with this topic not being covered in dentistry and optometry programs and by 62.5% of the PA programs. Lastly, schools were surveyed on certain social and cultural factors affecting pain. These results varied based on topic area (Cultural beliefs: Medicine 85.7%, APRN 83.3%, Physician Assistant 100%, Pharmacy 28.6%, Dentistry 50%, Optometry N/A; Age: Medicine 71.4%, APRN 66.7%, Physician Assistant 87.5%, Pharmacy 57.1%, Dentistry 50%, Optometry N/A). Differences were also noted in the proportion of schools that provided education on community program options to assist in the treatment of SUD with pharmacy and dentistry not covering the topic and only 50% of PA programs.

**Table 1:** Education on opioids and substance use disorders

Are students educated on/taught about…	Medicine N (%)	APRN N (%)	Physician assistant N (%)	Pharmacy N (%)	Dentistry N (%)	Optometry N (%)
DSM-5 criteria for Substance Use Disorder (SUD) as part of the core curriculum?	7 (100%)	5 (71.4%)	8 (100%)	6 (85.7%)	0 (0%)	0 (0%)
The neurobiology of SUD as part of the core curriculum?	7 (100%)	5 (83.3%)	7 (87.5%)	6 (85.7%)	0 (0%)	1 (100%)
The addiction cycle as outlined by Health and Human Services as part of the core curriculum?	7 (100%)	3 (50%)	5 (62.5%)	4 (57.1%)	0 (0%)	0 (0%)
The ethical issues in pain management?	7 (100%)	6 (100%)	8 (100%)	3 (42.9%)	2 (100%)	1 (100%)
The ethical issues in SUD?	4 (57.1%)	2 (33.3%)	5 (62.5%)	1 (14.3%)	0 (0%)	0 (0%)
Community program options to assist in the treatment of SUD?	3 (42.9%)	2 (33.3%)	4 (50%)	0 (0%)	0 (0%)	1 (100%)
The support infrastructure available to patients suffering with SUD?	6 (85.7%)	3 (50%)	6 (75%)	3 (42.9%)	0 (0%)	0 (0%)
The mechanism of pain in the human body (nociceptive pain, neuropathic pain, other pain?)	6 (85.7%)	5 (83.3%)	8 (100%)	7 (100%)	0 (0%)	1 (100%)
How to properly use patient pain assessment scales and tools?	6 (85.7%)	5 (83.3%)	8 (100%)	7 (100%)	1 (50%)	0 (0%)
Additional factors affecting pain: age?	5 (71.4%)	4 (66.7%)	7 (87.5%)	4 (57.1%)	1 (50%)	**---**
Additional factors affecting pain: cultural beliefs?	6 (85.7%)	5 (83.3%)	8 (100%)	2 (28.6%)	1 (50%)	**---**
Additional factors affecting pain: gender?	5 (71.4%)	3 (50%)	7 (87.5%)	4 (57.1%)	1 (50%)	**---**
Non-pharmacological methods for pain management?	4 (57.1%)	5 (83.3%)	8 (100%)	5 (71.4%)	1 (50%)	0 (0%)

The extent of education regarding pharmacological and non-pharmacological pain management is presented in Table 2. Medicine, APRN, physician assistant, and pharmacy schools had a “moderate” to “great” extent of pharmacologic therapy integration. Other pain management strategies were “somewhat” to “moderately” integrated.

**Table 2:** Extent of education

	Medicine Median (IQR)	APRN Median (IQR)	Physician Assistant Median (IQR)	Pharmacy Median (IQR)	Dentistry Median (IQR)	Optometry Median (IQR)
What is the extent of education students receive on the use of pharmacological therapy combinations for pain management?^a^	3 (2)	3.5 (--)	3 (--)	3 (--)	--	--
What is the extent of education students receive on the use of non-pharmacological therapy combinations for pain management?^a^	2 (0)	3.5 (--)	3 (2)	2.5 (--)	--	--
How much of the curriculum covers evidence-based plans for safe pain management in patients more susceptible to medication misuse?^a^	2 (1)	3 (2)	2.5 (--)	2 (1)	--	--
What is the extent of education students receive in managing chronic pain?^a^	2.5 (1)	3 (1)	2.5 (--)	3 (0)	--	--

^a^1=Not at all, 2= Somewhat, 3=Moderate, 4=Great

An examination of the integration of education on assessment, evaluation, and treatment is presented in Table 3. There were variations seen in training on risk of medication misuse based on various contributors to health (Disease History: Medicine 85.7%, APRN 83.3%, Physician Assistant 87.5%, Pharmacy 57.1%, Dentistry 50%, Optometry N/A; Culture: Medicine 14.3%, APRN 33.3%, Physician Assistant 50%, Pharmacy 28.6%, Dentistry and Optometry N/A). At least 667.7% of medicine, APRN, physician assistant, and pharmacy programs included motivational interviewing training, but dentistry and optometry did not.

**Table 3:** Education on assessment, evaluation, and treatment

Are students trained/educated/taught…	Medicine N (%)	APRN N (%)	Physician Assistant N (%)	Pharmacy N (%)	Dentistry N (%)	Optometry N (%)
To evaluate a patient using interviewing techniques such as motivational interviewing?	5 (71.4%)	4 (66.7%)	7 (87.5%)	6 (85.7%)	0 (0%)	0 (0%)
To evaluate a patient’s risk of medication abuse based on:						
Age?	4 (57.1%)	4 (66.7%)	7 (87.5%)	2 (28.6%)	1 (50%)	--
Culture?	1 (14.3%)	2 (33.3%)	4 (50%)	2 (28.6%)	--	--
Gender?	3 (42.9%)	2 (33.3%)	5 (62.5%)	2 (28.6%)	--	--
Disease history?	6 (85.7%)	5 (83.3%)	7 (87.5%)	4 (57.1%)	1 (50%)	--
How to search for a patient history of pain medication usage using Prescription Drug Monitoring Programs such as OARRS?	6 (85.7%)	3 (50%)	7 (87.5%)	5 (71.4%)	1 (50%)	1 (100%)
On the best practice methods to initiate and manage substance use disorder treatment?	5 (71.4%)	5 (83.3%)	8 (100%)	5 (71.4%)	0 (0%)	0 (0%)
In encounters involving high risk patients?^a^	6 (85.7%)	3 (50%)	4 (50%)	3 (42.9%)	1 (50%)	0 (0%)
In methods to safely taper pain medications?	5 (71.4%)	3 (50%)	6 (75%)	6 (85.7%)	0 (0%)	0 (0%)
How to support patients to avoid drug misuse?	5 (71.4%)	4 (66.7%)	7 (87.5%)	4 (57.1%)	1 (50%)	0 (0%)
How to support patients to avoid drug relapse?	3 (42.9%)	1 (16.7%)	5 (62.5%)	1 (14.3%)	1 (50%)	0 (0%)
On the steps to take when a patient has overdosed?	5 (71.4%)	4 (66.7%)	8 (100%)	6 (85.7%)	1 (50%)	0 (0%)
How to restart therapy after a relapse?	3 (42.9%)	0 (0%)	4 (50%)	3 (42.9%)	0 (0%)	0 (0%)
How to restart therapy after an overdose?	2 (28.6%)	0 (0%)	4 (50%)	2 (28.6%)	0 (0%)	0 (0%)
About the signs of substance misuse in chronic pain patient populations?	5 (71.4%)	4 (66.7%)	8 (100%)	6 (85.7%)	1 (50%)	1 (100%)

^**a**^ (High risk patients, according to the CDC< are those who exhibit some or all of the following: a past history of overdose, a history of substance use disorder, high opioid dosages (>50 MME/day), and concurrent benzodiazepine use.)

An entire section of the survey was dedicated to surveying schools about training in adverse childhood experiences related to pain management and treatment. Schools had variable rates of integration (childhood neglect: Medicine 42.9%, APRN 66.7%, Physician Assistant 25%, Pharmacy 14.3%, Dentistry and Optometry N/A; childhood exposure to domestic violence: Medicine 28.6%, APRN 66.7%, Physician Assistant 37.5%, Pharmacy 14.3%, Dentistry and Optometry N/A). Other information on the extent to which schools integrated education regarding ACEs can be found in Table 4.

**Table 4:** Education regarding adverse childhood events

Are students trained/educated/taught…	Medicine N (%)	APRN N (%)	Physician Assistant N (%)	Pharmacy N (%)	Dentistry N (%)	Optometry N (%)
To assess for childhood neglect in patients treated for pain?	3 (42.9%)	4 (66.7%)	2 (25%)	1 (14.3%)	--	--
To assess for childhood physical/psychological abuse in patients treated for pain?	3 (42.9%)	4 (66.7%)	5 (62.5%)	1 (14.3%)	--	--
To assess for sexual abuse as a minor in patients treated for pain?	3 (42.9%)	4 (66.7%)	3 (37.5%)	1 (14.3%)	--	--
To assess for childhood exposure to domestic violence in patients treated for pain?	2 (28.6%)	4 (66.7%)	3 (37.5%)	1 (14.3%)	--	--
To assess for parental psychopathology in patients treated for pain?	2 (28.6%)	3 (50%)	3 (37.5%)	1 (14.3%)	--	--
To assess for other traumatic childhood events in patients treated for pain?	2 (28.6%)	4 (66.7%)	4 (50%)	1 (14.3%)	--	--
Include steps to adapt a pain management plan based on adverse childhood events?	2 (28.6%)	0 (0%)	0 (0%)	1 (14.3%)	0 (0%)	0 (0%)

Schools were asked whether their students were educated in evaluating patients based on various social demographics that are related to the social determinants of health (Table 5). Schools results varied but remained below the 50% percent mark of schools that affirmed training in these social demographics. For example, education was more often discussed than insurance coverage and disability income support (Education: Medicine 42.9%, APRN 16.7%, Physician Assistant 62.5%, Pharmacy 28.6%, Dentistry and Optometry NA; Insurance Coverage: Medicine 28.6%, APRN 16.7%, Physician Assistant 62.5%, Pharmacy 25%, Dentistry and Optometry N/A; Disability Income Support: Medicine 28.6%, APRN 16.7%, Physician Assistant 50%, Pharmacy 14.3%, Dentistry and Optometry N/A).

**Table 5:** Education regarding the social determinants of health

Are students trained/educated/taught on evaluating patients based on…	Medicine N (%)	APRN N (%)	Physician Assistant N (%)	Pharmacy N (%)	Dentistry N (%)	Optometry N (%)
Sex	4 (57.1%)	4 (66.7%)	6 (75%)	3 (42.9%)	--	--
Race	3 (42.9%)	4 (66.7%)	6 (75%)	3 (42.9%)	--	--
Ethnicity	3 (42.9%)	4 (66.7%)	6 (75%)	3 (42.9%)	--	--
Marital status	4 (57.1%)	3 (50%)	4 (50%)	2 (28.6%)	--	--
Employment	3 (42.9%)	2 (33.3%)	5 (62.5%)	2 (28.6%)	--	--
Education	3 (42.9%)	1 (16.7%)	5 (62.5%)	2 (28.6%)	--	--
Insurance coverage	2 (28.6%)	1 (16.7%)	5 (62.5%)	2 (25%)	--	--
Disability income support	2 (28.6%)	1 (16.7%)	4 (50%)	1 (14.3%)	--	--
Geographical region	2 (28.6%)	2 (33.3%)	5 (62.5%)	1 (14.3%)	--	--

## Discussion

The results of the current survey of the healthcare professions in Ohio indicate that there are differences in the level and depth of pain management, SUD, and ACEs education provided. The President’s Commission on Combating Drug Addiction and the Opioid Crisis report promotes training all health care professionals in prevention, screening, identification, and treatment of OUD, a subset of SUD [19]. In conducting the present study, we set to establish a baseline for the level and extent of SUD training in a majority of the health professions. It is concerning that not all healthcare professionals who are able to prescribe opioids are educated on how to screen for OUD, the signs of SUD and how to use the PDMP. Here, we found that medical schools spend time on the teaching of the DSM5 criteria for OUD, the addiction cycle, and ethical issues surrounding SUD. On the other hand, less of an emphasis is placed on additional factors influencing pain and the perceptions of pain such as age, cultural beliefs, and gender. This is even more concerning as retrospective chart reviews found providers undertreat pain in patients who are older and in marginalized patient groups [16]. Pharmacy areas of focus tended to be in the areas of neurobiology and pharmacological management with very little emphasis on ethical issues associated with SUD. The dental and optometry programs had a greater focus in these latter areas. With the exception of optometry, none of the programs placed an emphasis in the area of training students on community program options for assisting in the treatment of SUD.

Several studies have reviewed the status of SUD education in the health care professions [24, 25]. The results of these database searches indicate a need for a unified interprofessional approach to SUD education [24]. Additionally, several discipline specific reports have suggested concerted effort needs in dental [1, 32], pharmacy [26], medicine [33] and PA [20, 38] training in these areas. In fact, several authors have suggested that interprofessional education, patient engagement, and OUD should be considered standards in SUD education [23, 25].

The over-prescribing of opioids has been associated with the onset of the first wave of the opioid epidemic [35]. There is, therefore, a critical need to provide adequate education to healthcare professionals in the area of SUD and OUD so as to attenuate this contributory role. A recent study found that physicians trained in “top-tier” medical school programs were less likely to prescribe opioids, suggesting a potential educational difference [30]. In this study, comprehensive data on all opioid prescriptions written by doctors in the United States between 2006 and 2014 were examined for the relationship between opioid prescribing and training. Schnell & Currie [30] found that Doctor of Osteopathic (DO) medicine prescribed more opioids than Doctor of Medicine (MD). Additionally, almost 50% of opioid prescriptions were written by general practitioners. From an educational perspective, Kolodny et al. [18] highlight the importance of prevention strategies, such as adopting the CDC prescribing guidelines and cautioning healthcare providers about prescribing opioids for both acute and chronic pain.

Unfortunately, many healthcare providers lack understanding regarding opioid risks, particularly the risk of addiction, and have an overestimation of opioid benefits. Kolodny et al. [18] conclude that “this pattern highlights the need for prescriber education explicitly correcting misperceptions about opioid pain relievers safety and efficacy.” Additionally, according to the 2019 Health Care’s Hidden Epidemic report, healthcare executives and providers cite a variety of tools that could help healthcare providers, including a more robust SUD education [12]. Adapting curricula to ensure providers can effectively recognize symptoms and risks of addiction will help address the rising opioid epidemic and improve patient outcomes [28].

In October 2019, the All-Ohio Medical School Opioid Use Disorder Collaborative reported on the development of a common medical school curriculum on pain management and OUD [2]. Of the seven medical schools in Ohio, following the meeting, two medical schools planned to add 11 topic areas to their curriculum and three schools plan to add three topic areas to their curriculum. Two schools did not state any plans to adjust their curriculum. The collaborative report noted the lack of participation by other practicing healthcare providers, other specialties, and non-clinical professionals.

Even with training on SUD and pain management, healthcare professionals can be unprepared to engage patients in the care process. In the area of SUD and pain management, patients should be engaged in the goal setting process and have realistic expectations for their care and the management of their pain or SUD. The CDC 2016 guidelines on chronic, non-cancer pain management outline the importance of patients being involved in goal establishment, risks and benefit assessment, and therapy management responsibilities. Patient engagement with shared decision-making and goal-directed encounters can improve health outcomes [8]. One patient-centered communication approach is motivational interviewing, as it allows the healthcare professional to engage in a goal-oriented encounter that is collaborative and caring [22]. Motivational interviewing is respectful of cultural differences and contributing factors, such as the social determinants of health, as the healthcare professional seeks to understand the patient’s perspective. This can be beneficial, given that many of the risks associated with SUD and pain management challenges are rooted in culture as well as the social determinants of health [13]. Of the respondents, over two-third of medicine, APRN, physician assistant, and pharmacy programs included motivational interviewing training. Schools should continue to incorporate this and other forms of patient-centered, goal-oriented communication strategies to address SUD, OUD and ACEs.

Including ACEs screening in healthcare practice provides the opportunity to improve health outcomes for patients, but this practice is not common. Reluctance to use ACEs screening tools may be due in part to perceived barriers. For example, ACEs may be viewed as psycho-social, or outside the expertise of primary care providers [36]. Additionally, providers may feel ill prepared to address any concerns uncovered in the screening process [17]. Identifying cases of trauma, and providing education, or treatment, can lower long-term health costs and support improved healthcare engagement for patients [11]. The significance of educating health care providers about ACEs, may also positively influence a long-term patient provider relationship. Higher ACEs have been associated with patient difficulty maintaining a long-term relationship with primary care providers [11]. Educating healthcare students is a primary strategy in supporting future prescribers in supporting the whole patient. Educating healthcare providers about ACEs should not stop at mere identification but should also include understanding ACEs and related impacts of trauma, incorporating trauma informed practices into daily treatment routines, and communication tools to support patients and their families. To date, there are no practice guidelines for ACEs and opioid prescribing and future work in this area is therefore warranted.

Strengths of this study include the response rates of several health professions programs in the state of Ohio. For example, medicine, pharmacy, dentistry, and optometry all had response rates of 100%. Thus, the information is representative of those health professions programs in Ohio. Furthermore, this is the first study to provide a comprehensive review of all the healthcare programs. However, a corresponding limitation is the response rate of other programs; APRN schools had lower response rates than other professions (27%) and may not be representative of all APRN programs in the state. Future exploration is warranted. The survey did undergo review before distribution and was built out of the literature; some items may not have been clear and could have been improved with pre-testing approaches, such as cognitive interviewing. Underpinning definitions were not outlined at the beginning of the survey; thus, respondents could have interpreted some of the definitions differently. The survey did undergo review prior to distribution, but the review process did not include significant pre-testing or validation. Another strength is that schools were directed to have the most appropriate person at the program complete the survey; however, it is unknown whether those individuals were the ideal individual. An additional limitation is that the survey was completed during the didactic portion of the students training and gaps identified may have been addressed in in residency training.

## Conclusion

The study finding suggests a need for uniform training requirements in the foundations of pain management, SUD, and ACEs. In addition to these core concepts, we suggest that an SUD curriculum should include the following elements:

1. Employ motivational interviewing techniques when evaluating pain and SUD patients
2. Evaluate social determinants of health while caring for patients in pain.
3. Assess the ethics behind treating patients with SUD.
4. Assess patients requiring pain managed based on ACEs.

From these findings, SCOPE is in the process of developing a statewide interprofessional educational symposium that target these areas of identified need.

## Data Availability

Availability of raw data and materials will be made available in accordance with the journal’s policy.

## Abbreviations

ACEs: Adverse childhood experiences
APRN: Advanced practice registered nurse
CDC: Centers for Disease Control and Prevention
MD: Doctors of Medicine
DO: Doctors of Osteopathic
IRB: Institutional Review Board
OUD: Opioid use disorder
PDMP: Prescription drug monitoring program
SCOPE: Scientific Committee on Opioid Prevention and Education
SUD: Substance use disorder
